# Workplace Incivility and Turnover Intention in Organizations: A Meta-Analytic Review

**DOI:** 10.3390/ijerph19010025

**Published:** 2021-12-21

**Authors:** Boshra H. Namin, Torvald Øgaard, Jo Røislien

**Affiliations:** 1 Norwegian School of Hotel Management, University of Stavanger, 4021 Stavanger, Norway; torvald.ogaard@uis.no; 2 Department of Quality and Health Technology, Faculty of Health Sciences, University of Stavanger, 4021 Stavanger, Norway; jo.roislien@uis.no

**Keywords:** workplace incivility, turnover intention, organizations, meta-analysis

## Abstract

Incivility has been identified as a prevalent and crucial issue in workplaces and one that may be associated with detrimental effects on employees and organizational outcomes, such as turnover intention. Many studies have been published regarding the effects of incivility, but there is a lack of integrative reviews and meta-analyses. The aim of the present study is to conduct an early meta-analysis of the relationship between employees’ perceptions of workplace incivility and their turnover intentions. Six databases, including ISI Web of Science, PsychInfo, Scopus, Emerald, Hospitality & Tourism Complete, and Soc Index, were searched to identify empirical articles for this meta-analytical paper. The results of statistical meta-analyses and meta-regression suggest that there is a positive relationship between perceived incivility and turnover intentions in employees and that relationship is consistent across different sources of workplace incivility. However, we did observe a possible interaction effect of “supervisor” and “coworker incivility”. The results also suggest that the relationship between workplace incivility and turnover intention is stronger in the academic sector than in other industries and stronger in the United States than in other countries.

## 1. Introduction

In recent decades, a distinct stream of research has focused on workplace incivility as a unique and lesser form of interpersonal mistreatment, which is prevalent and causes severe problems in various organizations [1,2,3,4,5,6,7,8,9,10,11,12]. Workplace incivility was first introduced in [12], which identified it by its ambiguity of intent and violation of workplace norms for mutual respect.

Workplace incivility generally encompasses recurrent rude and disrespectful behavior that violates mutual respect in the workplace with a low-intensity and unclear intent to harm the target [12], which is a widespread phenomenon in the working environment [13,14]. It has been reported that 98% of workers have experienced incivility and that half of them experienced it at least once a week [5]. The numbers have caused alarm as they reveal the serious impact of incivility on many employees and the resulting significant financial effects on organizations. Based on estimation in [15], cognitive distraction from work and project delays caused by workers being subjected to incivility lead to an annual cost of $14,000 per employee. In addition, employees who are the target of uncivil behavior in the workplace have to bear considerable human costs, such as emotional exhaustion [16], depression [17], and increased fear, sadness, and anger [18]. Moreover, lower organizational citizenship behavior [19], higher withdrawal behavior [5], turnover intention [20], and organizational exit [18] can all be behavior outcomes of employees who experience workplace incivility. Some studies also considered mediator or moderator variables in the relationship between perception of workplace incivility and turnover intention. For example, emotional exhaustion [1,21,22], job burnout [23,24], perceived organizational support [25], and job satisfaction [10,26] were considered as mediators, and Motherhood status [27], enactment [19], and role-ambiguity and team-building [28] were considered as moderators in that relationship

There is sufficient evidence to suggest that turnover intention is the immediate antecedent to real turnover behavior, which has become the main concern of service providers due to the higher costs this incurs [29,30]. A minimum of 5% of loss in total annual revenue is considered to be related to the cost of employee turnover [31]. A high level of employee turnover is closely related to a low level of organizational performance and productivity, which together result in rising costs of employee selection, recruitment, and training [31,32,33]. This clearly shows the important role of investigating antecedents and implementing strategies to reduce turnover intention in organizations.

### 1.1. Workplace Incivility

Workplace incivility is defined in [12] (p. 457) as “low-intensity deviant behavior with ambiguous intent to harm the target, in violation of workplace norms for mutual respect. Uncivil behaviors are characteristically rude and discourteous, displaying a lack of regard for others”. There is some overlap between workplace incivility and other negative treatments in the organization, including aggression, social undermining at work, deviance, antisocial behavior, violence [12]. However, they are different in their targets, intention to harm, continuation, intensity of the actions, and the type of norm violation, [11]. For example, the perpetrator of aggression has a clear intention to harm while incivility has an unclear intention that can be attributed to other factors, including the perpetrator’s personality, oversight, and ignorance, who can, in turn, claim that any harm done to target was accidental rather than intentional [12]. Different theories have been applied for aggression and workplace incivility in the literature. For example, attribution theory [34] and the script theory [35] for aggression and conservation of resource (COR) theory [36], and affective event theory [37] for workplace incivility. Only social learning theory [38] is evidenced to be used in both aggression and workplace incivility studies. In the management literature, rudeness refers to any insensitive or disrespectful behavior in the workplace, which may or may not be intentional, but even so, it violates social norms, and the target perceives it as rude [9,39]. Thus, rudeness can be referred to as incivility [40]. In general, any rude behavior in the workplace that is repeated over a period of time with low intensity that can be easily overlooked and has damaging effects at the individual, group, and organizational level [41] is regarded as workplace incivility. These behaviors are more verbal rather than physical, passive rather than active, indirect rather than direct, and subtle rather than overt. Examples are not saying “please” or “thank you”, spreading rumors, ignoring someone in a group, leaving rude messages, talking loudly about personal matters on the phone, taking credit for someone else’s efforts [11], blaming someone for no reason, and any body language or gestures that can be perceived as offensive. Given the behavior’s low intensity, the instigator of incivility can easily deny any such intention and may thus harm the target accidentally rather than intentionally.

By definition, incivility entails ambiguity and low intensity, but the effects can be quite severe. In fact, workplace incivility is considered to be one of the most harmful forms of mistreatment affecting employees in organizations [16], since employees are usually exposed to a series of emotion–cognition processes, including emotion evaluation (cognition) and cognition selection (response) [42]. An accumulation of unhealthy emotions in employees caused by workplace incivility may further lead to aggression and even trigger severe interpersonal conflicts [12]. This vicious cycle has the capacity to lead to serious negative effects on individuals and organizations [10,18,43]. Empirical evidence demonstrates that rudeness and uncivil behavior have negative effects on how individuals function at work, their creativity, work engagement, and their task performance ability [44,45]. 

Three main sources of incivility can exist within a work setting: customer, coworker, and supervisor incivility [14]. They are similar in context and definition but have different perpetrators; the perpetrators of supervisor and coworker incivility are internal, while the perpetrator of customer incivility is external to the organization [2]. Indeed, depending on the source of uncivil behavior and the preparators in the workplace, incivility would be perceived as differentially severe as others have argued (e.g., [44]). According to [46], many jobs in the service industry may be at risk in cases where there are multiple sources of incivility. This is especially true in relation to employees who are dependent on one another for providing customer services. 

According to the definition [12], different types/sources of incivility entail the same behavior but from different perpetrators inside or outside the organization [2,46]. In line with this argument, one may expect different sources of incivility to have a similar relationship with job outcomes and turnover intentions. Empirical studies have, however, revealed inconsistent results related to the strength of the relationship between different sources of workplace incivility and employees’ turnover intentions. Some studies have reported that supervisor incivility has a stronger relationship with turnover intention compared to coworker incivility [47,48,49,50], whereas other studies have shown that supervisor incivility and coworker incivility have a similar relationship with turnover intention, e.g., [7]. To the best of our knowledge, no study has considered customer incivility and its correlation with turnover intention compared to other sources of incivility. Moreover, the Workplace Incivility Scale (WIS) developed in [14] has been the most common measure of incivility in previous studies.

### 1.2. Turnover Intention

Turnover intention can be referred to as a “willingness to leave an organization” [51]. In fact, the intention of organizational members to quit their present job and look for other job opportunities because of dissatisfaction with their present job is referred to as turnover intention [52]. Based on this definition, turnover intention was used as a measure of the subjective feeling of organizational members regarding turnover rather than their specific behaviors [52]. According to [32], before making the final decision on turnover, employees usually go through a period of reflection to generate turnover. In this regard, turnover intention can be referred to as employees’ generation of the idea of turnover as well as their tendency to leave their present position and try to find another job because of their dissatisfaction [26]. Although many antecedents of turnover intention have been identified in previous studies, in a recent meta-analysis study [53], major antecedents were organized into nine categories, including work engagement (category: work attitudes), burnout (job strains), role conflict (role stressors), abusive supervision (supervisor and leader behaviors), deep acting (emotional labor), organizational citizenship behavior (performance), perceived organizational support (organizational contexts), and self-efficacy (individual differences). The current study focused on the antecedent role of workplace incivility, which is a form of job stress (job strains) according to the mentioned meta-analysis study [53].

Being the target or victim of uncivil behavior in the workplace is directly related to turnover intentions [14,43]. There is considerable evidence that in any individual who has faced workplace incivility, the incivility may be negatively related to job satisfaction, regardless of his/her perspective as a witness, instigator, or victim [10,13,14,17,41,48], which may result in a high turnover intention [10,17,54]. Workplace incivility may also lead to heavy work pressure for employees and generate instability and a high turnover intention in different industries [7,55,56].

One of the resource-based stress theories for understanding workplace incivility is the conservation of resource (COR) theory [36], which emphasizes the important role of valuable personal resources (i.e., objects, personal characteristics, or conditions) in individuals’ ability to deal with different stressors. Based on this theory, people are inclined to achieve, protect, and foster their valued resources in order to use them when encountering stressful interpersonal interactions, such as incivility [36]. COR theory asserts the fact that the valuable resources are limited and thus a deficiency in or loss of such resources could become challenging for the individuals who face new sources of stressors [36], and they may, in turn, show more negative job outcomes to compensate their resource loss. This theory has been mostly applied in cross-sectional incivility studies with a focus on one point in time (e.g., [2,21,24,28,57]). However, adaptation theory can identify the stressor–strain relationships explicitly over time [58]. Unlike COR theory, the notion of habituation in adaptation theory indicates that although an individual may be affected immediately and concurrently by a positive or negative stimulus in his/her life, such an effect should fade over time, and the person should return to present levels of well-being [59]. Based on this theory, it has been claimed in [20] that workplace incivility as an episodic stressor can be experienced again and again for a long time, and people may not only adapt themselves to but also systematically recover from experiencing that.

In a previous review paper [44], it has been suggested that conducting meta-analytic reviews of workplace incivility is required. The aim of this meta-analytic study is to answer two research questions: (a) How does the perception of workplace incivility affect employees’ turnover intention? and (b) Is this effect consistent if we check for different sources of workplace incivility (i.e., customer, coworker, and supervisor incivility), different workplace incivility measures, different industries, and different countries? Therefore, we hypothesized that the employees’ perceptions of workplace incivility have a positive relationship with their turnover intention and our overall assumption is that since possible effects of perceived incivility are a general phenomenon, the effects will be constant across sources of incivility, across different measures of incivility, different industries, and countries. The investigation starts with a systematic review of relevant literature related to workplace incivility and turnover intention and proceeds with a quantitative meta-analysis [60].

## 2. Methodology

### 2.1. Literature Search

This study adopted the method described by *the Cochrane Handbook for Systematic Review and Interventions* [61,62] for performing a meta-analysis of empirical studies investigating workplace incivility and turnover intention, along with *The Preferred Reporting Items for Systematic Reviews and Meta-Analyses* (PRISMA) *Statement* [60].

First, a systematic review was conducted, which needs sensibility and robustness in summarizing research [63]. The first literature search was conducted in spring 2019 in six electronic databases: ISI Web of Science, PsychInfo, Scopus, Emerald, Hospitality & Tourism Complete, and Soc Index, to identify empirical peer-reviewed articles that have been published in a 20-year period from 1999 (when workplace incivility was first introduced by Andersson and Pearson) to 2019. The selection of the databases was based on the coverage of social science, organizational behavior, and psychology. The following keywords were searched in various combinations: *uncivil behavior*, *organizational mistreatment*, *incivility*, *job outcome*, *customer*, *supervisor*, *coworker*, and *workplace*. This first search resulted in 658 papers. An additional search in Science Direct, Google Scholar, and ProQuest was conducted in summer 2019 to ensure other available articles were not missed. In order to strengthen the quality of the search, the searches were further refined using advanced searches and more specific and controlled search terms. The keywords used in this stage were “*workplace incivility*”, “*customer incivility*”, “*coworker incivility*”, “*supervisor incivility*”, “*employees’ outcome*”. The result from this additional search was 115 papers, resulting in a total of 773 papers from both searches.

In line with PRISMA 2009 statement, checking only the title and the abstract of all papers revealed 71 duplicated papers and 448 irrelevant papers. In line with the exclusion criteria (Table 1), the papers were eliminated if (1) they were review papers, research notes, book chapters, or unpublished dissertations, (2) they had inappropriate data including unsuitable variables, qualitative data, lack of measurement for incivility, and theoretical papers, and (3) they focused on incivility in contexts other than workplace incivility, such as public and criminal incivility, general cyber incivility, political incivility, family incivility, classroom incivility, etc. This excluded 519 papers.

In the next step, the remaining 252 papers were screened in detail, and 206 of them were excluded for not meeting the inclusion criteria (Table 1). Papers were excluded in this step if (1) they were not published in English, (2) were not in an organizational behavior context, (3) did not focus on at least one specific source of incivility (customer, coworker, supervisor incivility), (4) did not have an appropriate sample such as part-time or full-time employees in direct contact with supervisors, coworkers, and/or customers, and (5) did not investigate the relationship between incivility and employees’ outcomes.

**Figure 1:**
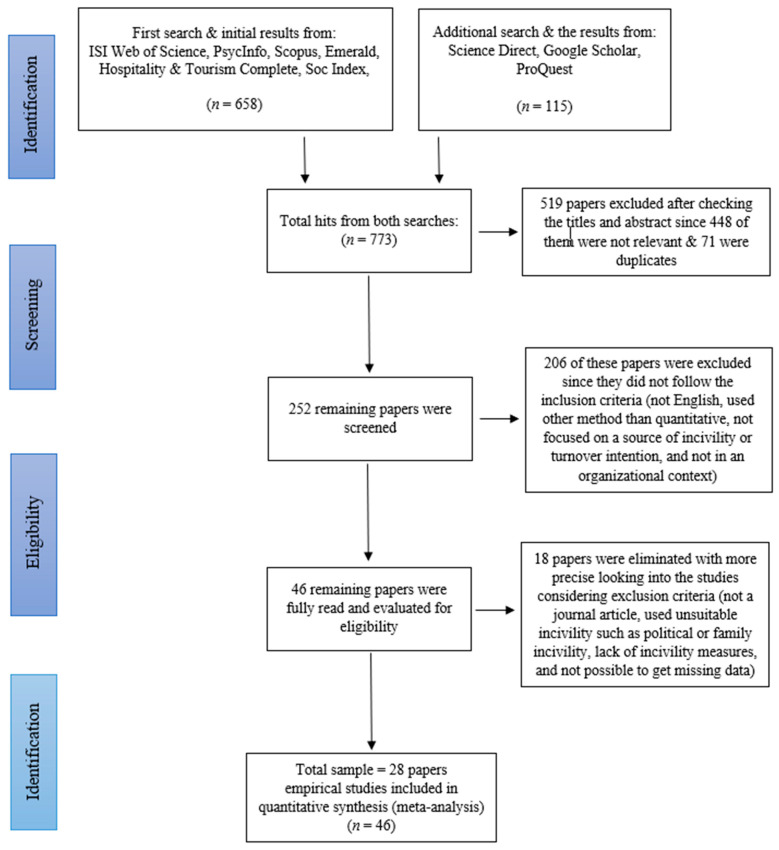
PRISMA 2009 flow chart.

The remaining 46 papers were carefully read in order to evaluate their eligibility, and a further 18 papers were eliminated: their main focus was on a different incivility context (i.e., cyber incivility, civility, tolerance for workplace incivility); they did not directly measure a source of workplace incivility or turnover intention (i.e., measuring counterproductive work behavior, negative work outcome); or they did not explicitly reveal necessary statistics, and we were unable to obtain those from the authors (see Figure 1). As a result, 28 papers were included in the final selection [1,6,7,10,17,19,20,21,22,23,24,25,26,27,28,49,50,57,64,65,66,67,68,69,70,71,72,73]. Some papers included two or three studies, some investigated the relationship between two different sources of incivility and turnover intention, and some compared findings in separate samples and over time. The final sample thus comprised 46 studies, as presented in Table 2. See Figure 1 for a flow chart of the process.

### 2.2. Data Evaluation and Statistical Analyses

**Figure 2:**
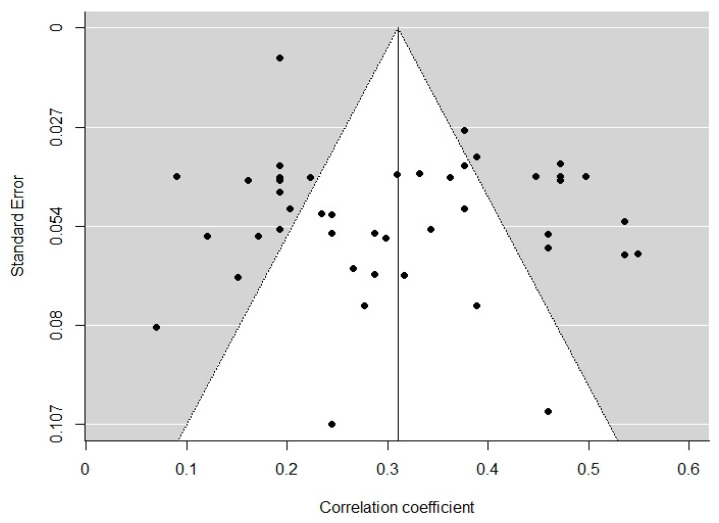
Funnel plot for illustrating publication bias.

A potential publication bias was first evaluated in a visual inspection of the funnel plot [74,75], see Figure 2. The points—each representing a single study—are evenly distributed on both sides of the summary effect size, indicating symmetry and hence, no bias. In order to further assess potential publication bias, we conducted a rank correlation test [76], which checks the relation between sampling variances and effect estimates for each study, and the alternative Egger’s regression test [77], which is more appropriate for smaller meta-analyses [76]. The results of both tests were statistically significant (*p* < 0.05), confirming that there is no significant publication bias present in the 46 studies.

The studies do not have functionally equivalent designs, and initial analyses of the heterogeneity of the effects suggested that the effects were not homogenous across studies (*I*^2^ = 89.59%, *p* < 0.001), all indicating that a random-effects model was appropriate [74,78]. Consequently, our analysis started with a random-effects meta-analysis including all 46 studies to evaluate our hypothesis.

In an attempt to identify sources of effect size variance, we proceeded with meta-regression moderation analyses [74,78]. First, we examined whether the three main sources of workplace incivility have different relationships to turnover intentions (customer, coworker, and supervisor incivility). Then we examined whether (1) the choice of incivility measures affects estimated effect sizes and whether the effect of workplace incivility on turnover intention (2) differs between industries and (3) countries.

Data were analyzed using the metafor package [74], which provides functions for conducting meta-analyses in R version 3.6.3 (R Core Team, Vienna, Austria) [79].

## 3. Results

### 3.1. Overall Effect

**Figure 3:**
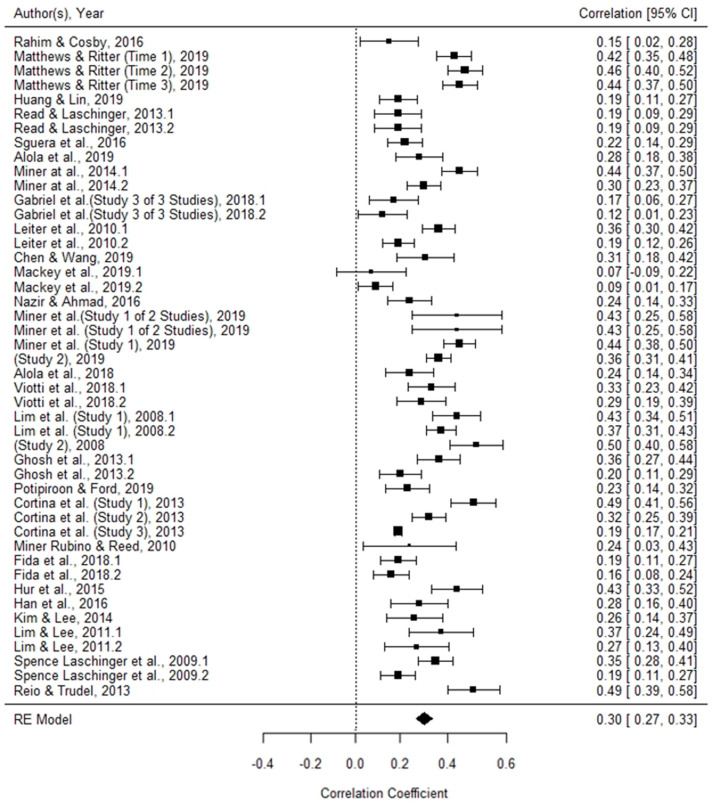
Forest plot for all studies included. Each point estimate (black square) bounded by a 95% CI, represents one study included in the meta-analysis. The black rhombus at the bottom of the plot represents the summary effect size and its width displays the 95% CI.

The data are summarized in Figure 3. The random-effects meta-analysis estimated an effect of workplace incivility on turnover intentions of (95% CI) 0.31 (0.26, 0.33), which was statistically significant (*p* < 0.001).

### 3.2. Sources of Incivility

The studies included were categorized into five groups according to the type of incivility reported. The majority of the studies (25) investigated “coworker incivility”, while “supervisor incivility” was reported in eight studies and “customer incivility” in three. The remaining 10 studies reported “supervisor *and* coworker incivility” (9) or “supervisor *or* coworker incivility” (1). Entering this into a meta-regression model as a categorical variable with five categories, and “coworker incivility” as the reference category, showed that the relationship between “supervisor incivility” and turnover intention was not statistically different to the relationship between “customer incivility” and turnover intention, compared to the baseline of “coworker incivility”. However, the combination of “supervisor *and* coworker incivility” did have significantly higher “turnover intentions” than the baseline (Table 3). However, the number of studies included in each of the categories was quite low, and results should be interpreted with caution. 

### 3.3. Incivility Measures

The popular measure of incivility (the Workplace Incivility Scale/WIS) [14] was used in 24 studies, while 9 studies used a modified or expanded version of WIS. In order to investigate whether the incivility measures make any difference to the results, we performed a meta-regression with measurement type as a categorical variable (WIS versus WIS-related measures). The estimated effect (95% CI) of using WIS-related measures rather than WIS was −0.04 (−0.12, 0.03) and was not statistically significant (*p* = 0.23). 

### 3.4. Industries

To explore whether the relationship between employees’ perceptions of workplace incivility and their turnover intentions differ among industries, we performed a meta-analysis with studies categorized according to four industry groups: healthcare (12 studies), academia (6), hospitality (4), and other sectors (24). The results indicated that for the academic sector, workplace incivility was associated with a higher turnover intention compared to the healthcare sector (Table 4)

### 3.5. Countries

Studies from the United States constitute almost half of the data included (19, 41.3%). In order to investigate if there is a difference between the United States and the rest of the world regarding the effect of workplace incivility on employees’ turnover intentions, we categorized studies into two groups. In line with the individualism–collectivism framework in [80], we comprised the US (North American) studies and all the other studies, respectively. A meta-regression showed a significantly smaller incivility-turnover relationship for the other countries compared to the United States, with an estimated smaller effect of −0.08 (−0.15, −0.005), *p* = 0.03.

## 4. Discussion

The aim of the present paper was to provide an early meta-analysis of the relationship between employees’ perceptions of workplace incivility on their turnover intentions. Analyses with a random-effects meta-analytic procedure [81] revealed that across the studies, there is a significant positive relationship between incivility and turnover intentions, supporting our hypothesis. Further comparisons of studies including either “coworker”, “supervisor” or “customer incivility”, respectively, did not reveal significantly different effect sizes. It is the most interesting finding that in studies with a combination of “coworker” and “supervisor incivility”, the effect on “turnover intentions” was lower than the sum of the direct effects in studies with only one of the sources. The effects were not additive, suggesting some form of interaction between the two sources. It has been evidenced that employees’ outcomes were less affected by coworker incivility compared to other sources of incivility in the workplace (e.g., [3,82]). Employees’ expression of negative emotions and their retaliation for uncivil behaviors from coworkers were perceived as less threatening than supervisor and customer incivility. Thus, the presence of coworker incivility may not lead to more resource depletion and surprisingly does not strengthen the negative effect of supervisor incivility on turnover intention. Considering the low-intensity characteristic of incivility and in line with the principles of adaptation theory, in [20], it has been argued that people may habituate to their negative emotions during and after experiencing incivility, and over time, they may return to their previous levels of well-being. This argument may also provide an explanation for our finding given that coworker incivility is also an internal stressor but perceived less risky than supervisor incivility, and its negative effect may fade during a long period. However, this novel finding needs to be more fully explored. 

In further meta-regression analyses of moderation effects of *incivility measures*, *industry*, and *country*, we found that there were no significant effect size differences between studies with different incivility measures. Furthermore, we found that only studies in the academic sector (six studies) reported significantly higher effect sizes (0.17, *p* = 0.005), while Effect sizes in the other sectors did not differ (not significant). Finally, we found that only studies from the United States reported significantly higher effect sizes than studies from other countries: effects were not statistically different in other countries.

Since the studies focus on perceived incivility, we should expect few differences in the effects between different types/sources of incivility [12]. Our results initially indicated that the effects of different types of workplace incivility indeed were not significantly different. However, we observed an interaction between “coworker” and “supervisor incivility”. This non-additive effect means that the simultaneous effect of coworker incivility and supervisor incivility on turnover intention is significantly less than the sum of the individual independent effects. The observed combined effect may be due to intercorrelation between coworker and supervisor incivility present in all relevant studies in our sample, implying that the individually estimated main effects of the two are overestimated. Furthermore, the observed effect may be related to ceiling effects in turnover intentions, with either the scale being too short to realistically capture the effects of the simultaneous coworker and supervisor incivility, or the respondents curbing their responses to the turnover intention scale. The observed effect may also be related to simple ceiling effects of the incivility perceptions, all of which would imply that our observed combined effect is underestimated. Finally, we may be observing the results of an underlying, dynamic, unobserved process in employees who feel exposed to simultaneous incivility from coworkers and supervisors.

The test for a moderation effect of incivility measures did not show any significant difference. This could be related to the close similarity between incivility measures used in our data. In fact, more than half of the studies included used the Workplace Incivility Scale [14] or a modified or extended version of this, by using different words for the same behavior, adapting the measurement items, referencing different sources/perpetrators of incivility, or soliciting incivility perceptions over different timespans (e.g., six months, one year, etc.). 

The result suggested that among industries, there is a stronger positive relationship between the perception of workplace incivility and the employees’ turnover intention in the academic work environment. This may be because the academic members are expected to show higher levels of respectful treatment, truthful relationships, and share knowledge with other members [83]. However, mistrusted relationship in academia and emphasis on competition leads to knowledge hiding behavior [84]. For instance, competition for pro-motions, titles, grant monies, and journal citations is common among faculties and faculty members [85]. Finally, knowledge hiding and competition may increase employees’ turnover intention [66,86]. Cultural tightness–looseness may also be relevant here since, in tight cultures with strong norms, there is little tolerance for deviant behaviors, whereas in loose cultures, there are weak norms and a high tolerance for such behaviors [87]. Nevertheless, the number of studies in industry categories was relatively limited, and especially all the studies in the academic sector were conducted in the US; thus, the results are tentative. The result also showed that the US (North American) employees’ perceptions of workplace incivility are more strongly related to their intentions to leave their jobs than employees from other countries. One possible explanation could be cultural differences. According to [80], the extent to which the members of a specific culture are able to control their desires and impulses is one of the influential dimensions used to classify that culture. Workplace incivility tends to be higher in “indulgent” cultures (e.g., Anglo-Saxon countries including the United States) that have weaker control over impulses compared with “restrained” cultures (e.g., Mediterranean countries) that have stronger control [80]. In an individualistic culture, such as the United States, the individual may feel more threatened by incivility and more challenged by the uncivil events since they may perceive incivility as an attempt to weaken their competitive strength [88]. In addition to different norms in responses to incivility across cultures, the tightness or looseness of a society (i.e., to what extent people may deviate from social norms) can affect their behaviors [87] as in a loose culture, for example, people are allowed for greater freedom and variety of responses to incivility [89].

## 5. Conclusions

This meta-analytical paper was an effort to provide a systematic review and integrate the results from previous studies about the relationship between employees’ perception of workplace incivility and their turnover intention. A significant positive relationship between workplace incivility and turnover intention was confirmed, and this result was consistent when we checked for different incivility measures and different sources of workplace incivility (i.e., customer, coworker, and supervisor incivility). Surprisingly, this positive relationship was not higher in the studies that considered a combination of two sources (supervisor and coworker incivility). Moreover, this was slightly higher in the academic sector compared to other industries, and it was also higher among the US (North American) employees compared to the employees from the category of other countries (i.e., five countries from different regions of Asia, one country from Europe, and one country from Africa). Although more studies are required, the results of this meta-analytical paper may provide sufficient insight into broad literature on workplace incivility as well as provide a basis for future research opportunities.

## 6. Limitation and Future Research

Similar to other meta-syntheses, the findings of our paper are limited by the quality of the studies and the original researchers’ interpretations. We searched nine databases to include as many studies as possible in order to obtain better primary meta-analysis results. Only published journal articles in English within organizational contexts were included in our sample. Thus, this study has a limitation of the language and the countries, especially WEIRD (Western, Educated, Industrialized, Rich, and Democratic) countries [90], which will affect the results and drawing conclusions. The sample was restricted to full-time or part-time employees who were exposed to and had perceptions of different sources of workplace incivility.

Although our findings are interesting, we need to caution against too comprehensive generalizations of the results. First, we must emphasize that the number of studies included is limited, and when we break the number of studies down into even smaller subsamples for the moderation analysis, the power of the analysis is quite small. Furthermore, we carried out multiple post hoc comparisons on the same data set with the standard 5% significance test, implying that the overall familywise significance will be lower than the standard 5%. Our results should therefore be taken as indicative and tentative rather than a final description. Furthermore, the strength of the relationship between incivility and turnover intentions varied between studies. Further studies will form a basis for more substantial conclusions and allow more penetrating analysis of the variance in effect sizes.

Our focus was on the relationship between incivility and only one outcome variable (i.e., turnover intention). Thus, for future research, we recommend studies of the relationship between workplace incivility and other employees’ job outcomes, such as well-being, affective commitment, burnout (e.g., [20]), psychological distress, and physical health (e.g., [66]), job satisfaction (e.g., [67]), organizational deviance and job performance (e.g., [25]), actual turnover, and eventually, further meta-analyses. The process in employees, starting with perceived incivility and ending in turnover intentions and eventual turnover, involves a long cause-and-effect chain of intervening emotional, cognitive, motivational, and physiological phenomena [20,25,66]. This lack of knowledge regarding the mediating mechanisms in existing empirical data emphasizes the need for closer scrutiny of the mentioned processes to deepen the understanding of the effects of incivility (e.g., [28,67]). Deeply penetrating explorative, qualitative, descriptive, and causal studies are much needed to investigate and identify the nature and dynamics of these cause-and-effect processes. 

The interesting non-linear effect of the combination of coworker and supervisor incivility offers great opportunities for future studies to investigate the interaction effects of multiple sources of workplace incivility and provide a nuanced and comprehensive understanding of their interplay and relative roles. Specifically, future research could benefit from contribution to both theoretical and practical implications. This can be achieved through extension to adaptation theory considering the role and effect of time by investigating repeated exposure to different sources of workplace incivility [20]. This should also inspire further research into the processes underlying the observed effect, as well as more studies evaluating the joint effect of all sources of workplace incivility on turnover intention. 

Since the majority of workplace incivility-related studies have been conducted in Western countries and the United States [91,92], and because cultural variation may affect employees’ perceptions and lead to variation in individual responses to incivility [93], knowledge building would benefit from more studies from the same culture as well as comparative, cross-cultural studies. Cross-cultural studies would guide generalizations across cultures [44] and could also shed light on whether differences in effects of incivility are due to cultural differences or differences in organizational policies and practices. Our result regarding the incivility–turnover relation across industries provides an important avenue for future research to explore such a relationship more fully in different industries, especially in the academic sector, where the role of working environment and culture seems to be very important in employees’ response to workplace incivility (i.e., turnover intention). Moreover, it could be interesting to investigate the relationship between the perception of incivility and turnover intention in the organizations with high versus low power distance culture and/or considering cultural tightness-looseness [94] to explore how employees interpret the treatment.

All studies included applied a common method (survey), and except for three studies [28,64,70], they are based on simultaneous measurement of both incivility and turnover intentions. The overall correlation will, therefore, most likely be inflated by common method variance [95]. Moreover, the survey/correlational design solely provides evidence of correlation with limited control for spurious correlations and relatively poor evidence for the actual causal flow. To establish more solid evidence for the causal flow, more time-series/time-lagged studies (e.g., [20,28,64,70]) and experimental designs are much needed. With more longitudinal studies using a different theoretical framework, such as adaptation theory (e.g., [20]), it would be possible to have more comprehensive meta-analytic studies in the future. Furthermore, well-planned and studied interventions aimed at changing the level of incivility in an organization and at empowering the employee to manage causes of incivility (e.g., [28,70]) are examples of applied studies that would be of great value to a practitioner in organizations with detrimental levels of incivility and provide insight into incivility-related processes and causal flows.

Even though our findings should be interpreted with the utmost caution, our analyses establish quite unequivocally, and across measures, types of incivility, industries, and countries, that there is a significant and substantial relationship between incivility and turnover intentions (e.g., [10,20]). Incivility thus warrants continued interest from practitioners as well as further research. This early meta-analysis shows that incivility is relevant and does have effects. We need additional studies to deepen our understanding of this negative factor in working life, to develop evidence-based recommendations for management and practice, and eventually, recommendations for public policy.

## Figures and Tables

**Table 1:** Inclusion and exclusion criteria.

Inclusion Criteria	Exclusion Criteria
Peer-reviewed articles published from 1999 to August 2019 Organizational behavior context Publication in English with quantitative design Papers including at least one source of incivility; customer, coworker, or supervisor incivility Studies considering the correlation between incivility and employees’ outcome (i.e., turnover intention) Samples with part-time or full-time positions who are in contact with managers/supervisors, coworkers, and/or customers (organizational context)	Unpublished dissertations, research notes, review papers, and book chapters Studies with inappropriate data (i.e., papers with qualitative data, unsuitable variables, lack of incivility measurement, and theoretical papers) Studies using incivility in contexts other than workplace incivility (i.e., political incivility, urban and social incivility, cyber incivility in general, school and classroom incivility, public and criminal incivility, family incivility, etc.) Studies for which it was not possible to get contact with the corresponding author(s) and obtain missing data

**Table 2:** Overview of studies included.

	Authors	Year	Journal	Country	Sample	Sample Size (*n*)	Correlation (r)	Type of Incivility	Incivility Measurement	Employees’ Outcome	Industry
**1**	Rahim and Cosby	2016	Journal of Management Development	U.S.	Employed undergraduate Business students + Colleagues+ Supervisors	223	0.15	Coworker Incivility	WIS	Turnover Intention	Other
**2**	Matthews and Ritter (Time 1)	2019	Journal of occupational health psychology	U.S.	Working adults	625	0.42	Supervisor or Coworker Incivility	WIS	Turnover Intention	Other
**3**	(Time 2)						0.46				
**4**	(Time 3)						0.44				
**5**	Huang and Lin	2019	Review of Managerial Science	Taiwan	High-tech and Banking Ind. employees	512	0.19	Coworker Incivility	WIS	Turnover Intention	Other
**6**	Read and Laschinger	2013	The Journal of Nursing Administration (JONA)	Canada	New graduate nurses	342	0.19	Supervisor Incivility	WIS	Job Turnover	Healthcare Industry
**7**							0.19	Coworker Incivility			
**8**	Sguera et al.	2016	Journal of Vocational Behavior	U.S.	Nurses working in a public research hospital	618	0.22	Coworker Incivility	Modified WIS	Turnover Intention	Healthcare Industry
**9**	Alola et al.	2019	Tourism Management Perspectives	Nigeria	Customer-contact employees in 4- and 5-star hotels	328	0.28	Customer Incivility	6 items from Cho, et al. (2016)	Turnover Intention	Hospitality Industry
**10**	Miner et al.	2014	Journal of Occupational Health Psychology	U.S.	Law school faculty members (Women)	594	0.44	Coworker Incivility	WIS	Turnover Intention	Academic work environment
**11**					Law school faculty members (Men)	640	0.3				
**12**	Gabriel et al. (Study 3)	2018	Journal of Applied Psychology	U.S.	Junior and senior undergraduate business students (Women)	319	0.17	Coworker Incivility	WIS	Turnover Intention	Other
**13**					Junior and senior undergraduate business students (Men)		0.12				
**14**	Leiter et al.	2010	Journal of Nursing management	Canada	Nurses	729	0.36	Supervisor Incivility	WIS	Turnover Intention	Healthcare Industry
**15**							0.19	Coworker Incivility			
**16**	Chen and Wang	2019	International Journal of Contemporary Hospitality Management	Taiwan	Tourist hotel chefs	226	0.306	Supervisor and Coworker Incivility	WIS	Turnover Intention	Hospitality Industry
**17**	Mackey et al.	2019	Journal of Business Ethics	U.S.	Manufacturing employees	156	0.07	Coworker incivility	Modified versionof Spector and Jex’s (1998) 4-item scale	Turnover Intention	Other
**18**					Full-time employees	620	0.09				
**19**	Nazir and Ungku	2016	International Review of Management and Marketing	Pakistan	Nurses in 10 selected healthcare settings	395	0.24	Supervisor and Coworker incivility	WIS	Turnover Intention	Healthcare Industry
**20**	Miner et al. (Study 1)	2019a	Equality, Diversity, and Inclusion: An International Journal	U.S.	Early-career STEM faculty (Women)	96	0.43	Coworker Incivility	WIS	Turnover Intention	Academic work environment
**21**					Early-career STEM faculty (Men)		0.43				
**22**	Miner et al. (Study 1)	2019b	The Journal of psychology	U.S.	Faculty members of different departments at a large university	742	0.44	Coworker Incivility	WIS	Turnover Intention	Academic work environment
**23**	(Study 2)				A nation-wide sample of Law School Faculty members	1300	0.36				
**24**	Alola et al.	2018	Sustainability	Nigeria	Customer contact employees of 4- and 5-star Hotels	329	0.24	Supervisor Incivility	Five items from Cho et al. (2016)	Turnover Intention	Hospitality Industry
**25**	Viotti et al.	2018	Journal of nursing management	U.S.	Nurses	341	0.33	Coworker Incivility	Four-item scale adapted bySliter et al. (2012)	Intention to Leave	Healthcare Industry
**26**				Italy		313	0.29				
**27**	Lim et al. (Study 1)	2008	Journal of applied psychology	U.S.	All employees of the Federal Courts of one of the larger circuits (Men)	325	0.43	Supervisor and Coworker Incivility	WIS	Turnover Intention	Other
**28**					All employees of the Federal Courts of one of the larger circuits (Women)	833	0.37				
**29**	(Study 2)				Employees of a midwestern municipality	271	0.5	Coworker Incivility	Expanded 12 items WIS		
**30**	Ghosh et al.	2013	Human Resource Development International	U.S.	Full-time employees from different organizations	420	0.36	Supervisor Incivility	Modified version of Reio’s (2011) based on WIS	Turnover Intention	Other
**31**							0.2	Coworker Incivility	Expanded 15 items WIS		
**32**	Potipiroon and Ford	2019	Journal of Occupational and Organizational Psychology	Thailand	Employees and their supervisors at a large public agency	401	0.23	Supervisor Incivility	WIS	Turnover Intention	Other
**33**	Cortina et al. (Study 1)	2013	Journal of Management	U.S.	City government municipality employees	369	0.49	Supervisor and Coworker Incivility	Expanded WIS	Turnover Intention	Other
**34**	(Study 2)				Law enforcement agency	653	0.32	Coworker Incivility	Expanded 20 items WIS		
**35**	(Study 3)				Military “active-duty members of the army”	15497	0.19		10 items from Aggressive Experiences Scale by Glomb and Liao (2003)		
**36**	Miner-Rubino and Reed	2010	Journal of Applied Social Psychology	U.S.	Employees of a property-management company	90	0.24	Supervisor and Coworker Incivility	Modified 8 items WIS	Turnover Intention	Other
**37**	Fida et al.	2018	Health care management review	Canada	Nurses	596	0.19	Coworker Incivility	The Straightforward Incivility Scale by Leiter & Day (2013)	Job Turnover	Healthcare Industry
**38**							0.16	Supervisor Incivility			
**39**	Hur et al.	2015	Human Factors and Ergonomics in Manufacturing and Service Industries	South Korea	Retail bank frontline employees	286	0.43	Coworker Incivility	Four items adapted from Sliter, et al. (2012)	Turnover Intention	Other
**40**	Han et al.	2016	International Journal of Hospitality Management	U.S.	Frontline service employees in independent Florida-based restaurants	228	0.28	Customer Incivility	14 items adopted from Burnfield et al. (2004)	Turnover Intention	Hospitality Industry
**41**	Kim and Lee	2014	Asian Women	South Korea	Women who work in sales service in clothing industry	239	0.26	Customer Incivility	Original scale developed by Wilson and Holmvall (2013)	Turnover Intention	Other
**42**	Lim and Lee	2011	Journal of Occupational Health Psychology	Singapore	Full-time employees from various organizations	180	0.37	Supervisor Incivility	Modified WIS	Intent to Quit	Other
**43**							0.27	Coworker Incivility			
**44**	Spence Laschinger et al.	2009	Journal of nursing management	Canada	Nurses	612	0.347	Supervisor Incivility	WIS	Turnover Intentions	Healthcare Industry
**45**							0.19	Coworker Incivility			
**46**	Reio and Trudel	2013	International Journal of Adult Vocational Education and Technology (IJAVET)	U.S.	Healthcare (143) + Manufacturing employees (127)	270	0.49	Supervisor and Coworker Incivility	WIS	Turnover Intention	Other

**Table 3:** Meta-regression analysis for the different incivility groups of the studies.

Type of Incivility	*n*	Estimate	95% CI		*p*-Value
Intercept/coworker incivility	25	0.27	0.23	0.32	<0.0001
Supervisor incivility	8	0.01	−0.08	0.11	0.745
Supervisor or coworker incivility	1	0.17	−0.05	0.40	0.136
Supervisor and coworker incivility	9	0.14	0.05	0.23	0.002
Customer incivility	3	0.005	−0.14	0.15	0.946

Notes: tau^2^ = 0.01, SE = 0.00, *I*^2^ = 86.69%.

**Table 4:** Meta-regression analysis for industry categories.

Type of Incivility	*n*	Estimate	95% CI		*p*-Value
Intercept/healthcare sector	12	0.24	0.18	0.31	<0.0001
Academic sector	6	0.17	0.05	0.29	0.005
Hospitality sector	4	0.03	−0.10	0.17	0.618
Other	24	0.07	−0.01	0.16	0.082

Notes: tau^2^ = 0.0121, SE = 0.0033, *I*^2^ = 87.12%.

## Data Availability

Not applicable.
